# Draft Genome Sequence of the Thermophilic Unicellular Cyanobacterium *Synechococcus* sp. Strain C9

**DOI:** 10.1128/mra.00294-22

**Published:** 2022-07-25

**Authors:** Megumi Kono, Joval N. Martinez, Takeshi Sato, Shin Haruta

**Affiliations:** a Department of Biological Sciences, Tokyo Metropolitan University, Hachioji, Tokyo, Japan; b Department of Natural Sciences, University of St. La Salle, Bacolod City, Negros Occidental, Philippines; c Department of Biological Sciences, Kanagawa University, Hiratsuka, Kanagawa, Japan; University of Delaware

## Abstract

This study presents the genome sequence of *Synechococcus* sp. strain C9 (= CCMEE 5213 = ATCC 700244), a thermophilic unicellular cyanobacterium that was originally isolated from a thermal pool at Octopus Spring, Yellowstone National Park, USA. The genome consists of a 2,958,309-bp chromosome with a GC content of 52.9% and 2,854 protein-coding sequences.

## ANNOUNCEMENT

*Synechococcus* is a polyphyletic unicellular cyanobacterial group (1, 2). *Synechococcus* sp. strain C9 was isolated from a thermal pool at Octopus Spring, Yellowstone National Park, USA (3), and grows at up to 55°C (4). Phylogenetic studies of the *Synechococcus* collective based on the 16S rRNA gene (5–7) indicated that strain C9 belonged to a deeply branching clade, the C9 lineage, which contains diazotrophic thermophiles such as *Synechococcus* sp. strain JA-3-3Ab and JA-2-3B′a(2–13) (8) and a nondiazotrophic mesophile, “*Brevicoccus berkleyi*” PCC 7336 (6). The C9 lineage is closely related to *Gloeobacter violaceus* PCC 7421, which has long been recognized as an ancient group in the phylum *Cyanobacteria* (9, 10), but is distantly related to a thermophilic group, the C1 lineage, containing *Synechococcus* C1 (3), *Synechococcus lividus* PCC 6715 (11), and “*Thermosynechococcus*” (6). Strain C9 has only 87 to 88% 16S rRNA identity to close relatives in the C9 lineage (7). Here, we report the genome sequence of strain C9 to contribute to the evolutionary and taxonomic studies of cyanobacteria.

Strain C9 (= CCMEE 5213 = ATCC 700244) was kindly provided by R. W. Castenholz. Strain C9 was grown in liquid BG-11 medium (12) at 55°C under fluorescent lamps at 30 μmol photon m^−2^ s^−1^. High-quality genomic DNA was extracted and purified by the Genomic-tip kit (Qiagen). The MGIEasy FS DNA library preparation set (MGI Tech) was used for the library preparation, and the genome was sequenced using the DNBSEQ-G400 platform (MGI Tech). A total of 13,986,408 reads with a 200-bp paired-end read length were sequenced using the DNBSEQ-G400RS high-throughput sequencing kit. Raw reads were trimmed using Sickle v1.33 (https://github.com/najoshi/sickle) with a minimum quality value score of 20 and a minimum nucleotide length of 127 nucleotides. The sequence assembly obtained using Unicycler v0.4.7 was further analyzed using KBase (13). Raw reads were assessed using FastQ v0.11.9 and reassembled by binning contigs using CONCOCT v1.1 (https://github.com/kbaseapps/kb_concoct). After assembly and quality filter binning using CheckM v1.0.18 (14), a total genome of 2,958,309 bp generated 4 contigs, with an *N*_50_ value of 2,835,982 bp, a GC content of 52.9%, and completeness of 98.3%. Automatic annotation was conducted using Prokka v1.14.5 (https://github.com/kbaseapps/ProkkaAnnotation) (15) and is available publicly in KBase Narrative (https://kbase.us/n/112786/3). Default settings were employed for all parameters.

The assembled genome of *Synechococcus* sp. strain C9 consists of 2,854 protein-coding sequences, 44 tRNAs, and 1 rRNA cluster. The 16S rRNA gene sequence was 100% identical to the sequence reported previously (5). Average nucleotide identity by BLAST (ANIb) values for strain C9 with other members in the C9 lineage were 65 to 67%. The genome of strain C9 contains the gene sets for molybdenum-iron nitrogenase (*nifXNE* and *nifBSUHDKVZT*). The diazotrophic members in the C9 lineage have *nifT* in the *nif* operon (8), but BLAST searches using their *nifT* genes as queries found no homologous gene in strain C9; the functions of the NifT protein remain unknown (16).

### Data availability.

The whole-genome shotgun project has been deposited in DDBJ/ENA/GenBank under the accession number JALAAD000000000. The version described in this paper is the first version, JALAAD010000000. The raw sequence reads are available in the SRA database under the accession number SRR18210225. All project data are available under the BioProject accession number PRJNA811380.
